# Patients with high-dose diuretics should get ultrafiltration in the management of decompensated heart failure: a meta-analysis

**DOI:** 10.1007/s10741-019-09812-2

**Published:** 2019-06-17

**Authors:** Xiaofeng Shi, Jiating Bao, Haili Zhang, Hao Wang, Lei Li, Yue Zhang

**Affiliations:** 1 Emergency department, Tianjin First Center Hospital, Tianjin, China; 2 Intensive Care Unit, Tianjin First Center Hospital, Tianjin, China; 3 General Surgery Department, West China Hospital of Sichuan University, Chengdu, Sichuan China; 4 Department of Vascular Surgery, The Second Hospital of Dalian Medical University, Dalin, China; 5 Institute of Urology, The second Hospital of Tianjin Medical University, Tianjin, China

**Keywords:** Ultrafiltration, Diuretics, Decompensated heart failure, Serum creatinine, Meta-analysis

## Abstract

The identification of specific patients with decompensated heart failure (DHF) who may benefit from ultrafiltration (UF) is important in clinical practice. We undertook a meta-analysis to compare the effects of ultrafiltration and diuretics on major clinical outcomes. The outcomes included weight change, length of hospital stay, rehospitalization for HF, mortality, change in serum creatinine, dialysis dependence, and adverse outcomes. We identified 14 trials including 975 patients with HF, met the eligibility criteria. There was a reduction in heart failure-related rehospitalization in ultrafiltration group when compared with the diuretic group. Subgroup analyses revealed a trend toward the decreased HF readmissions in ultrafiltration plus diuretic therapy group but did not reach statistical significance compared with ultrafiltration alone therapy. Overall, UF treatment did not produce apparent beneficial effects for weight loss, lengths of hospitalization, total mortality, the change of serum creatinine, and dialysis rate. Subgroup analyses showed increase in the serum creatinine were significantly higher for a higher dose regimen (> 200 mg/day) when compared with lower dose diuretic therapy (< 200 mg/day). As for adverse events, UF patients were associated with an increased risk of hypotension and lower risk of neurologic symptoms. The current results revealed ultrafiltration was associated with significant reduction in the rate of rehospitalization. Increase in the serum creatinine was observed in patients with high-dose diuretic regimen. Patients with high-dose diuretics should get ultrafiltration therapy.

**Electronic supplementary material:**

The online version of this article (10.1007/s10741-019-09812-2) contains supplementary material, which is available to authorized users.

## Introduction

Decompensated heart failure (DHF) has caused rising concerns of general public over these years. DHF is the common cause for hospitalization and emergency visit among medicare beneficiaries [1, 2]. Most HF patients went into emergency department with symptoms of volume of overload and abrupt onset of dyspnea. Traditional therapy of patients with DHF was diuretics which induced a rapid diuresis that reduced congestion and dyspnea [3, 4]. However, these drugs may cause acute kidney injury, abnormal neurohormonal activation, and electrolyte imbalance, and there is an urgent need to develop alternative treatment strategy that will favorably alter deadly condition.

Ultrafiltration as an alternative method is used to improve volume overload symptoms in all subsets of HF patients, including those with diuretic resistance or renal insufficiency [5–7]. The ability to precisely control the removal of sodium and water allows the ultrafiltrate extracted from serum during UF therapy to be isotonic. Many randomized, controlled trials (RCTs) have compared the efficacy and safety of ultrafiltration with pharmacologic therapy including UNLOAD (the Ultrafiltration versus Intravenous Diuretics for Patients Hospitalized for Acute Decompensated heart Failure) study [8], CARRESS-HF (Cardiorenal Rescue Study in Acute Decompensated Heart Failure) study [9], and AVOID-HF (Aquapheresis Versus Intravenous Diuretics and Hospitalization for Heart Failure) trial [10]. However, the results from the reported trials were inconsistent, leading to uncertainty about the effects of UF. The previously published systematic reviews that evaluated the efficacy of UF in treatment of patients with decompensated heart failure lacked appropriate safety evaluation or did not include all related trials [11, 12]. There are still unanswered questions regarding whether ultrafiltration should combined with diuretic therapy, best types of ultrafiltration, the optimal rate of filtration, and the optimal dose of loop diuretics.

We therefore undertook a meta-analysis to compare the efficacy and safety of UF with diuretic therapy for decompensated heart failure patients.

## Methods

### Data sources, search strategy, and selection criteria

Relevant studies were identified by searching Medline via Ovid (from 1950 to December, 2018), Embase (from 1966 to December, 2018), and the Cochrane Library database (Cochrane Central Register of Controlled Trials), with relevant text words and medical subject headings that included “heart failure”, “ultrafiltration”, “clinical trial”. Trials were limited to randomized controlled trials (RCTs) without language restriction. Reference lists from identified trials and review articles were searched manually to identify any other relevant studies. We also searched the Clinical Trials.gov website for relevant trials that were registered as completed but not yet published. We performed a systematic review of the published articles in terms of the approach recommended by the guidelines for the conduct of meta-analyses of intervention studies.

### Data extraction and quality assessment

Published literatures were obtained from each eligible trial, and relevant information was extracted into a spreadsheet. The extracted data included patient age, serum creatinine, ejection fraction, inclusion criteria of patients, diuretics dose, and duration of ultrafiltration. The literature search, data extraction, and quality assessment (Grading of Recommendations Assessment, Development and Evaluation system) [13] were undertaken independently by two authors (Xiaofeng Shi and Jiating Bao) using a standardized approach. Any disagreement in extracted data was adjudicated by a third reviewer (Yue Zhang).

### Outcomes

The outcomes included weight change, length of hospital stay, rehospitalization for HF, mortality, change in creatinine, dialysis dependence, and adverse outcomes.

### Statistical analysis

The odd risk (OR) and 95% confidence interval (CI) for each outcome were calculated before pooling by the random effects model. For the continuous variables, we used the weighted mean difference between groups. The percentage of variability across studies attributable to heterogeneity beyond chance was estimated with the *I*^2^ statistic. Potential publication bias was assessed with the Egger test and represented graphically with Begg funnel plots of the natural log of the OR versus its standard error (SE). A two-sided *p* value less than 0.05 was regarded as significant for all analyses. All statistical analyses were done with STATA (version 13.0) and Review Manager 5.0.

## Results

### Trial flow and characteristics of included studies

**Fig. 1:**
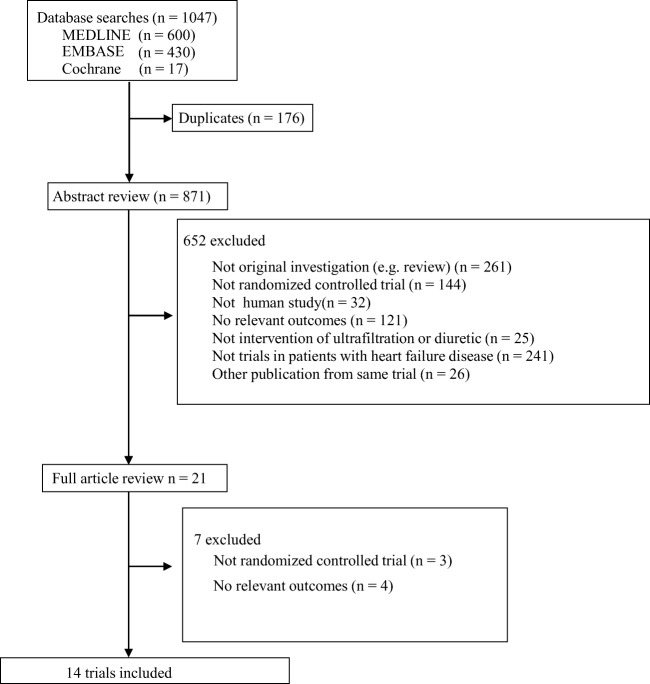
Identification process for eligible studies

The literature search yielded 1047 articles, of which 21 were reviewed in full-text. A total of 14 trials including 975 patients with HF met the inclusion criteria in our study (Fig. 1) [8–10, 14–24]. The mean age ranged from 56 to 75 years and follow-up ranged from 12 h to 180 days. The UF group patients in four studies were randomized to UF combined with diuretics therapy, while in other seven trials, the UF group patients used UF therapy alone. The characteristics of the included studies are summarized in Table 1.

**Table 1:** Characteristics of included studies

Study	Country	Inclusion criteria	Number of patients (ultrafiltration/diuretics)	Ultrafiltration intevention	Duration of ultrafiltration	Control intevention	Diuretics dose (mg/day)	Mean age (years)	Serum creatinine (mg/dl)	EF (%)
Agostoni 1994	Italy	Chronic HF	8/8	UF plus furosemide	NA	Furosemide	100.00	58.50	NA	23.00
AVOID-HF 2016	USA	Acute HF	110/111	UF	80 h	Furosemide	271.00	67.00	1.5	36.00
Badawy 2012	Egypt	Acute HF	20/20	UF	72 h	Furosemide	500.00	64.00	1.40	< 40%
CARRESS-HF 2012	USA	Acute HF and cardiorenal syndrome	94/94	UF	40 h	Diuretics	120.00	65.00	2.00	33.00
Chung 2014	USA	Acute HF	8/8	UF	NA	Furosemide	212.00	69.00	1.90	24.00
CUORE 2014	Italy	HF	27/29	UF plus furosemide	19 h	Furosemide	153.00	75.00	1.70	32.00
Hanna 2011	USA	Acute HF	19/17	UF	22 h	Diuretics	NA	60.00	1.70	19.00
Pepi 1993	Italy	HF	12/12	UF plus furosemide	NA	Furosemide	NA	56.50	NA	24.00
RAPID-CHF 2005	USA	Acute HF	20/20	UF plus furosemide	8 h	Diuretics	160.00	68.00	1.70	< 40%
Seker 2016	Turkey	Acute HF	10/20	UF	20.5 h	Furosemide	164.00	66.50	1.56	31.00
Shen 2017	China	HF	65/65	UF	8 h	Furosemide	NA	57.50	NA	NA
Tabakyan 2010	Russian	Chronic HF	19/21	UF	NA	Diuretics	> 80	62.00	1.40	32.00
ULTRADISCO 2011	Italy	Acute HF	15/15	UF	46 h	Furosemide	250–500	68.00	2.10	32.00
UNLOAD 2007	USA	ADHF	100/100	UF	12.3 h	Diuretics	181.00	62.50	1.50	< 40%

*HF*, heart failure; *UF*, ultrafiltration; *NA*, not available

### Quality of trials

Key indicators of trial quality were analyzed by modified Jadad quality scale system, including the process of randomization, concealment of allocation, and the use of intention-to-treat analysis (Table 2).

**Table 2:** Quality assessment

Study	Sequence generation	Allocation concealment	Blinding	Incomplete outcome data	Selective outcome reporting
Agostoni 1994	Unclear	Unclear	No	Yes	No
AVOID-HF 2016	Yes	Unclear	No	Yes	No
Badawy 2012	Unclear	Unclear	No	Yes	No
CARRESS-HF 2012	Yes	Unclear	No	Yes	No
Chung 2014	Unclear	Unclear	No	No	No
CUORE 2014	Yes	Unclear	No	Yes	No
Hanna 2011	Unclear	Yes	No	Yes	No
Pepi 1993	Unclear	Unclear	No	Yes	No
RAPID-CHF 2005	Yes	Unclear	No	Yes	No
Seker 2016	Unclear	Unclear	No	No	No
Shen 2017	Unclear	Unclear	No	Yes	No
Tabakyan 2010	Unclear	Unclear	No	No	No
ULTRADISCO 2011	Yes	Unclear	No	No	No
UNLOAD 2007	Yes	Unclear	No	Yes	No

### Weight loss

**Fig. 2:**
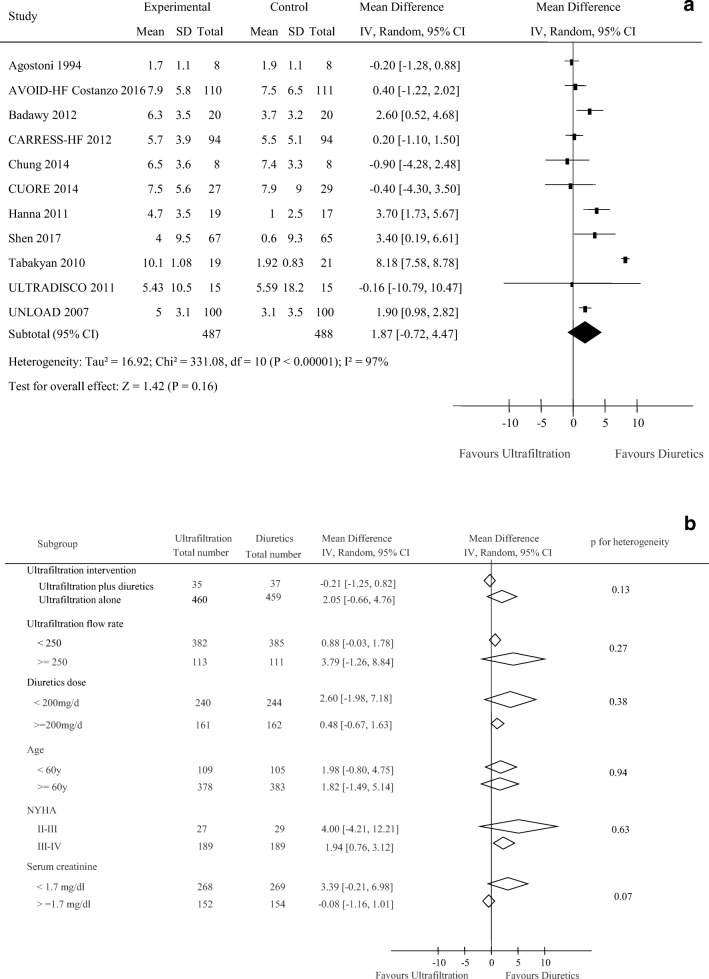
Pooled weight loss (kg) (**a**) and subgroup analysis of weight loss (**b**)

Data regarding the effects of UF on weight loss were available from 12 trials including 991 participants. As shown in Fig. 2a, UF treatment did not produce an apparent beneficial effect for weight loss (weighted mean difference, 1.65 kg [95% CI, − 0.83 to 4.14 kg], *p* = 0.19; *I*^2^ = 97%, *p* < 0.001). Subgroup analyses were performed for the weight loss (Fig. 2b). No clear evidence of heterogeneity was found in comparisons of summary results obtained from subsets of studies grouped by ultrafiltration intervention, ultrafiltration flow rate, diuretics dose, patients’ age, NYHA classification, and serum creatinine level (all *p* > 0.05).

### Lengths of hospitalization and rehospitalization for HF

**Fig. 3:**
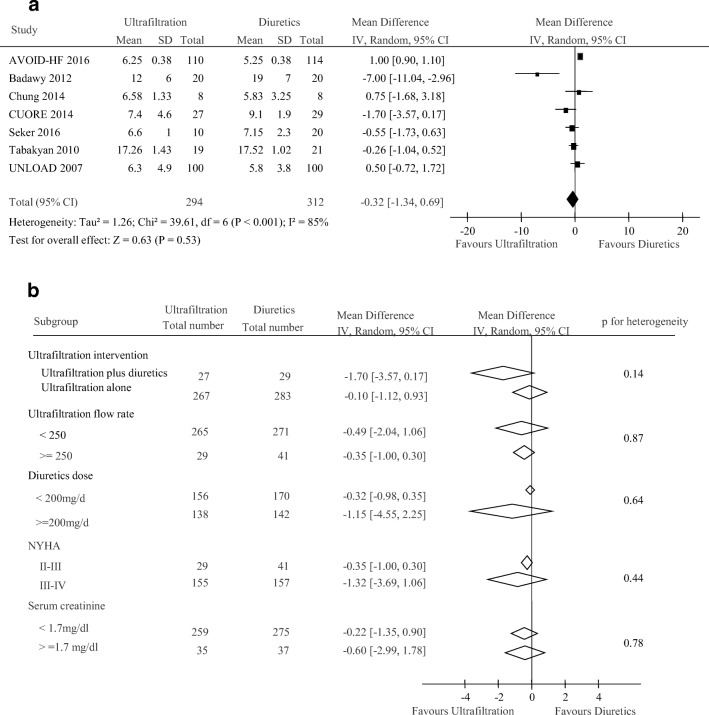
Pooled lengths of hospitalization (day) (**a**) and subgroup analysis of lengths of hospitalization (**b**)

**Fig. 4:**
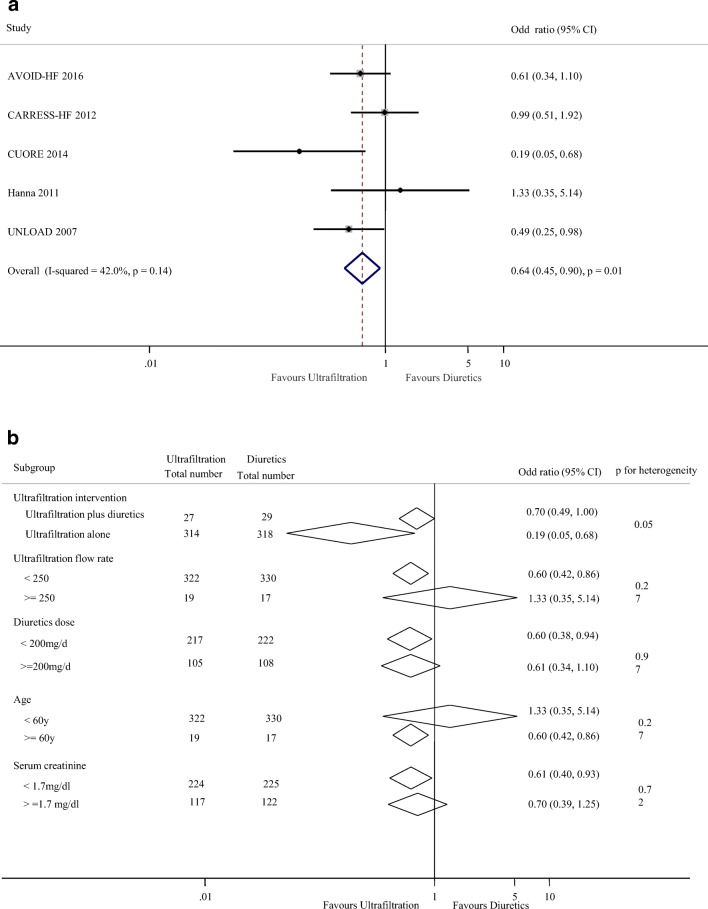
Pooled rehospitalization for heart failure (**a**) and subgroup analysis of rehospitalization for heart failure (**b**)

We next compare the efficacy of UF with diuretic therapy on lengths of hospitalization and rehospitalization for HF. Length of hospitalization was reported in 7 studies with 606 patients. There was no significant difference in lengths of hospitalization (weighted mean difference, − 0.32 days [95% CI, − 1.34 to 0.69 days], *p* = 0.53; *I*^2^ = 85%, *p* < 0.001, Fig. 3a) between this two groups. Subgroup analyses showed there were no clear evidence of heterogeneity in comparisons of summary results obtained from subsets of studies grouped by ultrafiltration intervention, ultrafiltration flow rate, diuretics dose, age, and NYHA classification (all *p* > 0.05, Fig. 3b). In terms of rehospitalization, five studies reported 78 events in 341 patients with UF treatment (22.8%) and 111 events of the 347 patients with diuretics therapy (31.9%). There was a reduction in heart failure-related rehospitalization in ultrafiltration group when compared with the diuretic group (OR 0.64; 95% CI, 0.45 to 0.9, *p* = 0.01; *I*^2^ = 42%, *p* = 0.14, Fig. 4a). We noted a different magnitude of effect according to the ultrafiltration intervention in trials; the OR was 0.70 (95% CI, 0.49 to 1.00) for ultrafiltration plus diuretic therapy compared with 0.19 (95% CI, 0.05 to 0.68) for ultrafiltration alone therapy (*p* for heterogeneity = 0.05). There was no apparent heterogeneity of effect between trials grouped by ultrafiltration flow rate, diuretics dose, age, and serum creatinine (all *p* > 0.05, Fig. 4b).

### Total mortality

**Fig. 5:**
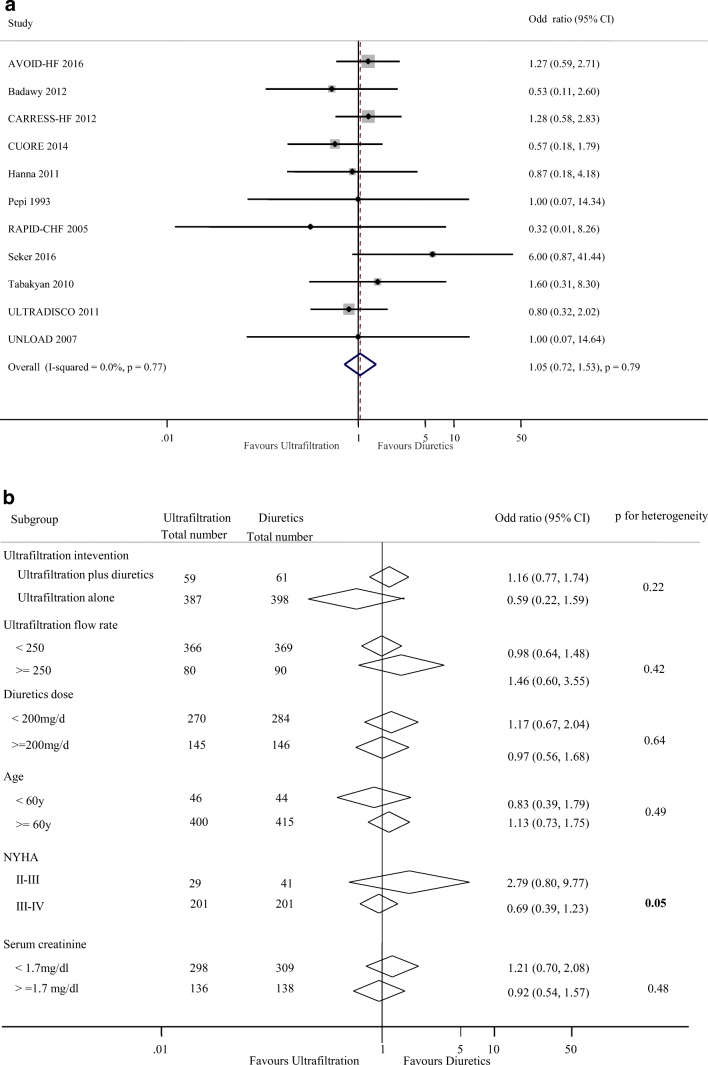
Pooled total mortality (**a**) and subgroup analysis of mortality (**b**)

Eleven studies reported 65 deaths in 447 patients with UF treatment (14.5%) and 63 deaths of the 460 patients with diuretics therapy (13.6%). Overall, UF therapy did not reduce total mortality of HF patients (1.05; 0.72 to 1.53, *p* = 0.79) as compared with diuretics therapy with no heterogeneity (*I*^2^ = 0.0%; *p* = 0.77, Fig. 5a). No clear evidence of heterogeneity was found in comparisons of summary results obtained from subsets of studies grouped by ultrafiltration intervention, ultrafiltration flow rate, diuretics dose, age, NYHA classification, and serum creatinine (all *p* > 0.05, Fig. 5b).

### Changes of serum creatinine and dialysis dependence

**Fig. 6:**
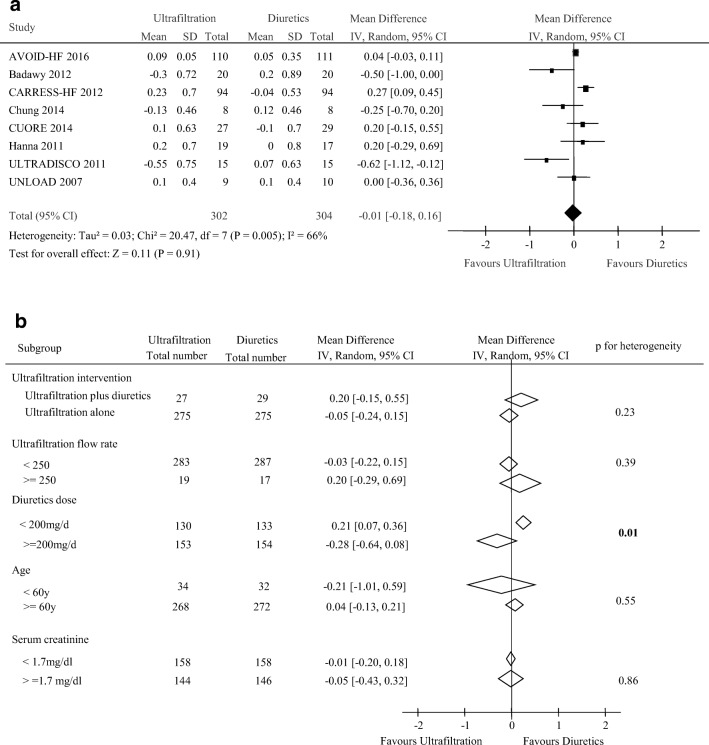
Pooled the change in serum creatinine (mg/dl) (**a**) and subgroup analysis of the change in serum creatinine (**b**)

**Fig. 7:**
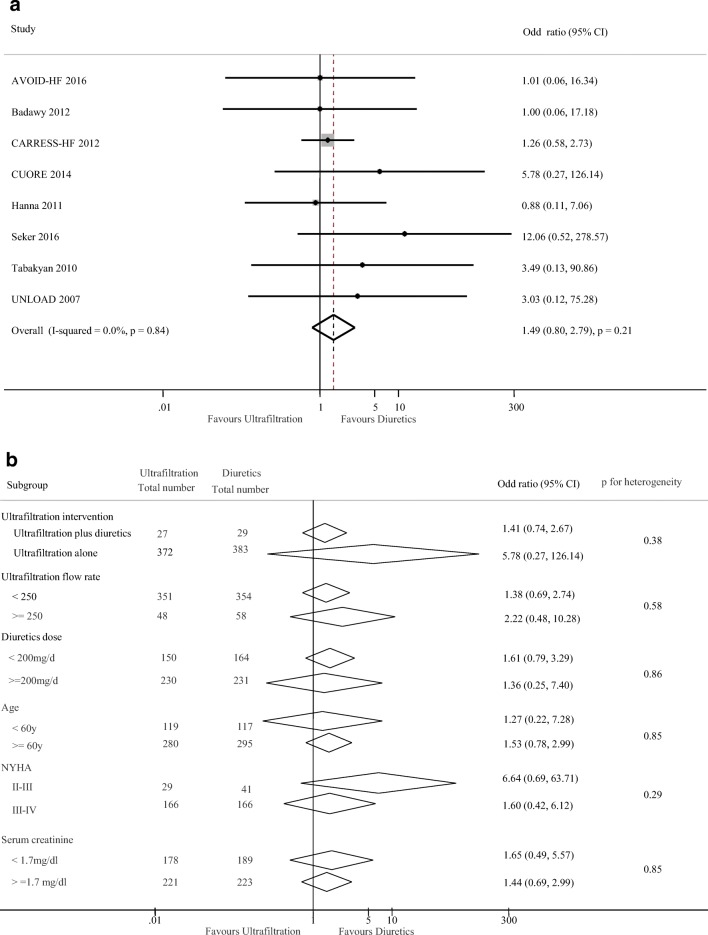
Pooled dialysis dependence (**a**) and subgroup analysis of dialysis dependence (**b**)

Eight trials including 606 participants reported the change of serum creatinine and eight studies reported 45 dialysis patients out of 811 total patients. There were no difference seen in the change of serum creatinine (weighted mean difference, − 0.01 mg/dl [95% CI, − 0.18 to 0.16 mg/dl, *p* = 0.91; *I*^2^ = 66%, *p* = 0.005, Fig. 6a) and dialysis rate (1.49, 0.80 to 2.79, *p* = 0.21; *I*^2^ = 0%, *p* = 0.84) between the two groups (Fig. 7a). Subgroup analyses showed a different magnitude of effect according to the diuretic dose used in trials; the OR was − 0.28 (95% CI, − 0.64 to 0.08) for a higher dose regimen (> 200 mg/day) compared with 0.21 (95% CI, 0.07 to 0.36) for lower dose therapy (< 200 mg/day) (*p* for heterogeneity = 0.01, Fig. 6b). Subgroup analysis for the effect of UF on dialysis was seen in Fig. 7b. No significant heterogeneity was found in these studies grouped by ultrafiltration intervention, ultrafiltration flow rate, diuretics dose, age, NYHA classification, and serum creatinine (all *p* > 0.05, Fig. 7b).

### Adverse events

Date on adverse outcomes were reported by a few trials, including worsening HF, cardiovascular outcome, hemorrhage, infection, hypotension, anemia or thrombocytopenia, electrolyte disorder, neurologic, filter clot, cerebral circulation disturbance, emergency department visits, and mechanical ventilation (Table 3). Six trials provided data for hypotension. UF patients were associated with an increased risk of hypotension (2.39; 1.20 to 4.76, *p* = 0.01). Only two studies reported the events of neurologic symptoms and showed UF therapy was associated with a lower risk of neurologic symptoms (0.35; 0.13 to 0.93, *p* = 0.04), which limited the power of difference due to small sample size. There were no differences noted in the incidence of other adverse events between the two groups (all *p* > 0.05, Table 3).

**Table 3:** Adverse events reported in the included RCTs

Adverse event	Total trial	Events/ultrafiltration	Events/diuretics	OR (95% CI)	*p* value
Worsening HF	5	74/251	101/249	0.58 (0.26, 1.14)	0.11
Cardiovascular outcome	7	119/380	139/381	0.70 (0.32, 1.49)	0.35
Hemorrhage	5	15/333	13/342	1.19 (0.30, 4.76)	0.80
Infection	6	21/353	15/366	1.46 (0.65, 3.27)	0.35
Hypotension	6	28/271	13/284	2.39 (1.20, 4.76)	0.01
Anemia or thrombocytopenia	2	11/194	5/194	1.63 (0.55, 4.78)	0.38
Electrolyte disorder	1	0/94	3/94	0.14 (0.00, 2.72)	0.19
Neurologic	2	6/210	16/211	0.35 (0.13, 0.93)	0.04
Filter clot	2	7/127	0/129	8.35 (1.00, 69.24)	0.05
Cerebral circulation disturbance	1	1/19	0/21	3.49 (0.13, 90.86)	0.45
Emergency department visits	3	37/213	43/211	1.07 (0.31, 3.70)	0.92
Mechanical ventilation	1	1/20	2/20	0.50 (0.05, 5.08)	0.56

*RCT*, randomized controlled trials; *HF*, heart failure; *OR*, odd ratio

### Publication bias

Begg’s funnel plot and Egger’s test suggested there was no evidence of publication bias for the outcome of rehospitalization (*p* = 0.81, Supplementary figure 1).

## Discussion

UF is a therapy full of promise, but has yet to find a definitive role. In this large quantitative systematic review comprising 14 trials and 975 individuals, we demonstrated UF therapy reduced HF-related hospital admissions compared with diuretic therapy. There was no evidence of any difference in weight loss, the length of hospitalization, and mortality rate. The changes of serum creatinine and dialysis rate were similar in both groups. Notably, increase in the serum creatinine was significantly higher for a higher dose regimen (> 200 mg/day) when compared with that of lower dose diuretic therapy (< 200 mg/day). There was an increased frequency of episodes of hypotension and a decreased frequency of neurologic symptoms in the UF group. These results suggested UF appears to be an efficacious therapy, but should be used with caution in HF patients.

ACCF/AHA guideline for the management of heart failure recommends ultrafiltration may be considered for patients with refractory congestion not responding to medical therapy [25, 26]. The quality of the evidence was generally low (2C). The question of whether acute heart failure will benefit from ultrafiltration at an early stage remains unresolved. In recent years, many studies compared the effectiveness and safety of diuretics versus ultrafiltration for the treatment of HF. The UNLOAD trial is the first landmark trial in this field [8]. The results showed ultrafiltration had a more pronounced effect on weight loss and fluid removal than diuretics therapy, and was associated with a decrease in rehospitalisation for 200 congested patients with AHF. One major shortcoming of this study was that the better outcomes in the UF group could be attributed to more complete decongestion. In a subsequent study, CARRESS-HF, conducted in 188 patients with AHF and worsening renal function, showed UF led to a worsening in renal function with no significant difference in weight reduction between the two groups [9]. Patients experienced more adverse events in the ultrafiltration group compared with diuretics group. The major shortcoming was that it had no measures in place to ensure optimal volume depletion in the UF group. The rate of fluid removal was mandated to be 200 ml/h, which might be excessive for patients with hypotension and greater dependence on preload for hemodynamic stability. Recently, the AVOID-HF trial was terminated early when 224 of the 800 planned patients with AHF had been enrolled [10]. The preliminary data showed the UF group trended toward a longer time to first HF event within 90 days and fewer HF and cardiovascular events; also, more patients in the UF arm experienced adverse events. In AVOID-HF, the average UF rate of 138 ml/h was lower than 200 ml/h rate of the CARRESS-HF trial, and therapy was delivered over a longer period (70 h vs. 41 h, respectively). However, similar to the UNLOAD trial, fluid removal was greater in the UF group, which would result in similar beneficial findings in AVOID-HF trial. Actually, in today’s evidence-based world and pragmatic trials world, there was insufficient evidence to state one therapy over the other. A meta-analysis on ultrafiltration in acute heart failure by Waqas et al. demonstrated that ultrafiltration has advantages in fluid removal, weight loss, and reduction in heart failure rehospitalization [27]. Another review by Kwok et al. reported a consistent reduced rehospitalization effect of ultrafiltration compared with diuretics but no differences in weight loss, length of hospitalization, and mortality [12]. Consistent with the results of Kwok et al., our analysis showed ultrafiltration treatment was associated with a reduction in the rate of rehospitalization for heart failure. Congestion is recognized as a major cause for rehospitalization in patients with DHF. It is conceivable that fluid removal could have a salutary impact on the rate of rehospitalization. There was a trend toward the greater weight loss in ultrafiltration group in our analysis. Current study suggested that UF should be considered for management of patients with DHF; however, whether the high upfront cost of UF therapy would be offset by reduction in the rate of HF-related rehospitalization and resource utilization is yet unknown. This is an important point determining whether ultrafiltration should be routine used at early stage in HF while not as second-line treatment.

Although Kwok et al. published the most recent review, they did not analyze the adverse events. While intravenous diuretics were supposed to contribute to worsen renal function, no different effects on renal failure or creatinine changes between ultrafiltration and diuretics were observed in our study. Some other adverse effects were common in both groups, an increased frequency of hypotension was observed for ultrafiltration group and a higher risk of neurologic symptoms in diuretic arm. Other adverse events such as cardiovascular outcome, hemorrhage, or emergency department visits were not increased overall. Hypotension is commonly encountered in clinical practice. UF should be adjusted to suit the circumstances of each patient with lower blood pressure and greater dependence on preload for hemodynamic stability.

The challenge of our study was interpreting the findings in view of subgroup analysis. For a long period, diuretics have been used as the usual care of heart failure; however, the effectiveness often declined with repeated exposure of diuretics [3, 28]. Use of ultrafiltration in HF has been shown to increase diuretic responsiveness. A question worth exploring is whether ultrafiltration plus diuretic therapy is superior to ultrafiltration alone. A trend toward the decreased HF readmissions in ultrafiltration plus diuretic therapy group was observed but did not reach statistical significance compared with ultrafiltration alone therapy. Diuretic agents were stopped after randomization in many studies. Therefore, future studies should be designed to state whether ultrafiltration plus diuretic therapy is superior to ultrafiltration alone. Next, the efficacious and safe diuretic dose used in trials was needed to be determined. We found these was no significant difference between different doses diuretic (furosemide dose > or < 200 mg/day) concerning weight loss and lengths of hospitalization, nor in rehospitalization for HF. As for safety endpoint, we noted increase in the serum creatinine was significantly higher for a higher dose regimen (> 200 mg/day) when compared with lower dose diuretic therapy (< 200 mg/day). Worsening renal function has been associated with a strongly increased mortality in heart failure. Current practice guidelines suggested patients with a degree of diuretic resistance should get UF treatment. However, it was recently reported that worsening renal failure alone is not an independent determinant of the outcomes in patients with AHF [29]. Testani et al. recently showed no increase in urinary biomarkers indicative of tubular damage during diuretic therapy in the ROSE-AHF trial [29]. However, Akihiro et al. pointed out such patients who did not have an adverse outcome may have “pseudo-WRF” [30]. The prognosis might be different depending on the mechanism of renal dysfunction in HF. Therefore, interpretation of the impact of UF on the change of serum creatinine could prove challenging until we are able to better characterize renal function in the setting of HF. The clinical impact of diuretic dose in patients with ADHF was also explored. Peacock et al. analyzed data from the ADHERE registry including 82,540 patients with ADHF to compare the clinical and renal outcomes associated with lower versus higher loop diuretic dose (< 160 mg vs. ≥ 160 mg of furosemide) [31]. The results showed patients receiving the higher doses of loop diuretic had a higher risk for in-hospital mortality, instances of worsening renal function, and prolonged hospitalization. Therefore, in this study, we support ultrafiltration as a bail-out therapy for patients with adequate diuretic therapy (> 200 mg/day).

The study has some potential limitations. First, we found evidence of substantial heterogeneity in outcomes, although we tried to address this by using random effects model and subgroup analysis. We acknowledge the possibility that this heterogeneity had an impact on our results. Second, as the target populations of this meta-analysis were heart failure patients, the urgency of the disease and the seriousness of the consequences decided the moderate number and size of trials. Last, there is lack of detailed diuretic protocol available in most trials.

## Conclusions

The current results revealed ultrafiltration was associated with significant reduction in the rate of rehospitalization but not provided significant benefit on weight loss, length of hospitalization, and mortality. Increase in the serum creatinine was observed in patients with high-dose diuretic regimen. Physicians should take into consideration that patients with high-dose diuretics should get ultrafiltration therapy.

## Electronic supplementary material

ESM 1 (PPTX 17 kb)
