# Supplementary figures and images for: Patients with high-dose diuretics should get ultrafiltration in the management of decompensated heart failure: a meta-analysis
Source: Heart Fail Rev. 2019 Jun 17;24(6):927–40. doi: 10.1007/s10741-019-09812-2 (PMC6834743; doi:10.1007/s10741-019-09812-2)

## Slide 1
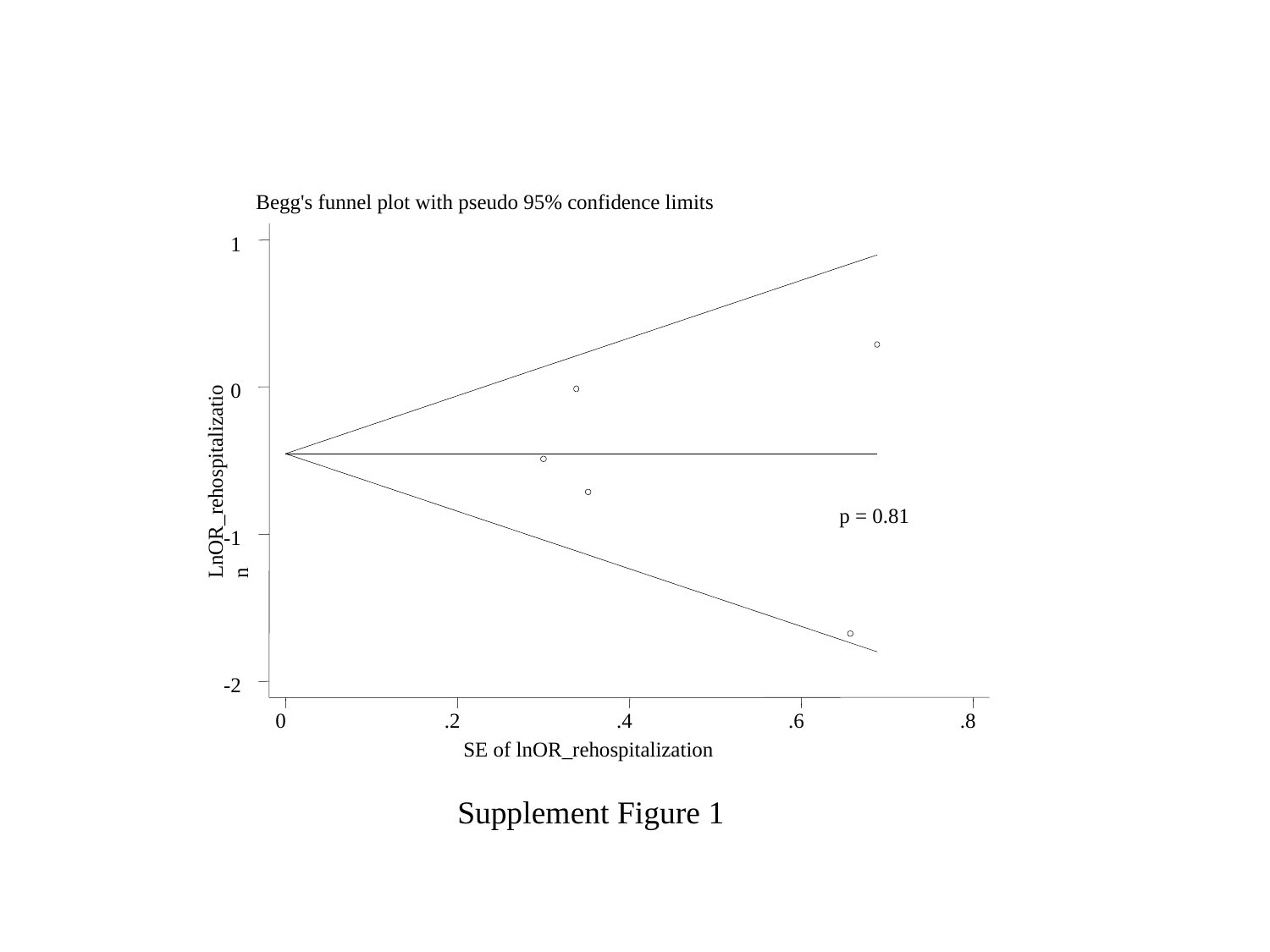

Supplement: Supplementary file 1 — (PPTX 17 kb) [file 10741_2019_9812_MOESM1_ESM.pptx]
